# Structure and Dynamics of Inhomogeneities in Aqueous Solutions of Graft Copolymers of N-Isopropylacrylamide with Lactide (P(NIPAM-*graft*-PLA)) by Spin Probe EPR Spectroscopy

**DOI:** 10.3390/polym14214746

**Published:** 2022-11-05

**Authors:** Ekaterina M. Zubanova, Tatiana A. Ivanova, Evgenii A. Ksendzov, Sergei V. Kostjuk, Peter S. Timashev, Mikhail Ya. Melnikov, Elena N. Golubeva

**Affiliations:** 1 Faculty of Chemistry, Lomonosov Moscow State University, 119991 Moscow, Russia; 2 Research Institute for Physical Chemical Problems of the Belarusian State University, 220006 Minsk, Belarus; 3 Institute for Regenerative Medicine, Sechenov First Moscow State Medical University, 119992 Moscow, Russia; 4 Faculty of Chemistry, Belarusian State University, 220006 Minsk, Belarus; 5 N.N. Semenov Federal Research Center for Chemical Physics, Russian Academy of Sciences, 119991 Moscow, Russia

**Keywords:** thermoresponsive polymers, electron paramagnetic resonance, nitroxides, inhomogeneities, coil-to-globule, spin probe

## Abstract

Coil-to-globule transition and dynamics of inhomogeneities in aqueous solutions of graft copolymers of NIPAM with different content of oligolactide groups were studied using spin probe continuous wave EPR spectroscopy. The technique of the suppressing of TEMPO as spin probe by spin exchange with Cu^2+^ ions was applied. This approach allowed us to detect individual EPR spectra of the probe in collapsed globules and estimate its magnetic and dynamic parameters reliably. The formation of inhomogeneities at temperatures lower than the volume phase transition temperature measured via transmission, and differential scanning calorimetry was fixed. An increase in oligolactide content in copolymers leads to the formation of looser globules, allowing for the exchange of the probe molecules between the globules and the external solution.

## 1. Introduction

Thermoresponsive polymers pique the interest of researchers, owing to their controllable behavior in solutions when responding to changes in temperature [1,2]. Miscibility, solubility or swelling degree may change when passing critical temperature. For example, poly-N-isopropylacrylamide (PNIPAM) and its copolymers undergo coil-to-globule phase transition in aqueous solutions [3]. Below lower critical solution temperature (LCST), these polymers are soluble in water due to the formation of hydrogen bonds between water molecules and hydrophilic amide groups in the polymer chains. Upon heating above LCST, hydrogen bonds degrade, and hydrophobic interactions prevail, leading to the collapse of the polymer coil into a dense globule. LCST is frequently measured via differential scanning calorimetry (DSC) or turbidimetry [3]. In these cases, the observed critical temperature is often listed as volume phase transition temperature (VPTT) [4] and cannot account for the formation of nanoscopic inhomogeneities in polymer solutions. Such inhomogeneities in solutions of thermoresponsive polymers may be observed with dynamic light scattering [5] and some probe methods [6].

The temperature-controlled properties of PNIPAM allow for its different biomedical applications, e.g., in delivery for drug systems [7,8], biomolecules [9,10] and protein separation [11], as well as in tissue engineering as smart hydrogels [12] and coatings for cell dishes [13,14]. In the latter case, PNIPAM coatings allow for 2D- and 3D-cell structures with developed extracellular matrices because of spontaneous detachment of cell structures from the thermoresponsive coatings or scaffolds due to polymer swelling or solvation during the globule-to-coil transitions [15,16]. In aqueous solutions, PNIPAM has a sharp phase transition with LCST of 305 K [17], resulting in the fast nondestructive elimination of the cell sheet from the PNIPAM surface. In order to vary the LCST values and increase cell adhesion, copolymers of N-isopropylacrylamide were synthesized and tested. Additionally, NIPAM-based copolymers with N-tert-butyl acrylamide (NTBA) [18] and the recently synthesized series of graft copolymers of PNIPAM with oligolactide (PLA) [5] were reported. The insertion of aliphatic polyester groups into the polymer chain increases its biocompatibility and biodegradation [19]. The degradation of P(NIPAM-*g*-PLA) scaffolds due to the hydrolysis of the oligolactide molecules may increase LCST during the cell growth process, resulting in the polymer scaffold dissolving at physiological temperatures [20]. This finding expands the scope of possible applications of P(NIPAM-*g*-PLA) in biomedicine as injectable hydrogels for the formation of 3D cell structures [21,22,23].

Spin probe electron paramagnetic resonance (EPR) spectroscopy has proven itself quite useful in studying polymers [24,25]. EPR spectra of spin probes as tracer molecules evolve with the changes in their environment; this is largely in response to the changes in the polarity of solutions, as well as the mobility of polymer chains. For the solutions of thermoresponsive polymers, spin probes may be captured by collapsing globules during phase transitions. This process manifests itself in the EPR spectra [6,26]. The (2,2,6,6-Tetramethylpiperidin-1-yl)oxyl, a stable, small amphiphilic nitroxyl radical, commonly known as TEMPO, is often used to study hydrophilic/hydrophobic polymer systems. Decreasing the environment polarity in polymer globules causes changes in the spin Hamiltonian parameters of the spin probe, specifically affecting a_N_, the tensor of hyperfine coupling (hfc) on the paramagnetic ^14^N nuclei [25]. The a_N_ of the spin probe in the hydrophobic polymer globule is lower than that in aqueous solutions, which is manifested in the splitting or broadening of the components in the EPR spectra. The changes in spin probe mobility are characterized by isotropic rotational correlation time t_corr,iso_ (the average time required for the rotation of a molecule at one radian). This parameter governs the shape of the EPR spectra for nitroxide radicals. The spin probe captured by the polymer aggregate rotates slower than in solutions and has a broadened EPR signal. The emerging polymer aggregates (i.e., inhomogeneities) may have different rigidity, free volume or polarity depending on the polymer composition and structure. Kurzbach et al. applied the spin probe technique to study the influence of polymer composition on the nature of nanoscopic inhomogeneities in aqueous solutions of different copolymers of polyethylene oxide and polypropylene oxide and defined two types of inhomogeneities [27]. For copolymers with a larger content of hydrophobic blocks, the EPR spectra and, consequently, the measured paramagnetic parameters of the spin probe in the polymer globule do not change with temperature. This is attributed to the formation of rigid globules or inhomogeneities referred to as static. On the other hand, in the solutions of polyethylene oxide copolymers with small hydrophobic blocks, dynamic inhomogeneities may form. They arise when tracer molecules are exchanged between the inhomogeneities and the external solutions. Since the external solution (usually water-based) is more polar than the polymeric globule, the exchange of probe molecules increases the a_N_ value. Therefore, the dynamic inhomogeneities manifest themselves in a high-field shift of the EPR spectra for the probe in the globule.

In this paper, we studied coil-to-globule transitions and the dynamics of inhomogeneities in aqueous solutions of graft copolymers of NIPAM with different content of oligolactide groups via CW EPR spectroscopy. We suppressed the TEMPO EPR signal via spin exchange with Cu^2+^ ions [28,29]. This technique allows for the detection of individual EPR spectra of the probe in the collapsed globule while measuring its paramagnetic and dynamic parameters reliably [26]. As a result, the formation of inhomogeneities at temperatures lower than LCST measured via transmission and differential scanning calorimetry (DSC) was fixed.

## 2. Materials and Methods

### 2.1. Substances

**Figure 1:**
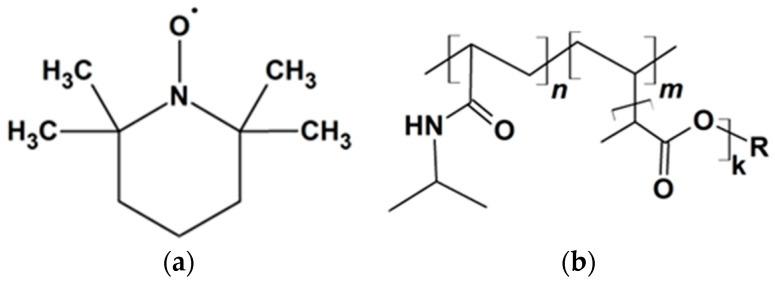
Structures of radical (2,2,6,6-tetramethylpiperidin-1-yl)oxyl (TEMPO) (**a**) and graft copolymers P(NIPAM-*g*-PLA) (**b**).

The stable (2,2,6,6-tetramethylpiperidin-1-yl)oxyl radical (TEMPO) (see Figure 1a) and copper (II) chloride dihydrate CuCl_2_·2H_2_O purchased from Sigma-Aldrich (Burlington, MA, USA) were used without further purification. A total of 0.01 M phosphate-buffered saline (PBS), pH = 7.4, was prepared by dissolving the tablets from Puschinskiye Laboratorii (Pushchino, Russia) in distilled water. Thermoresponsive graft copolymers P(NIPAM-*g*-PLA) (see Figure 1b) with different content of oligolactide (3, 9, 17 wt%) were synthesized by RAFT (reversible addition−fragmentation chain transfer)-mediated copolymerization of N-isopropylacrylamide (NIPAM) and oligolactide bearing methacrylic end group using 2-(dodecylthiocarbonothioylthio)-2-methylpropanoic acid (DMP) as the RAFT agent [5]. Methacrylate-terminated oligolactide macromonomers with number average molar masses of 600 and 1200 Da were used in copolymerization with NIPAM, referred to as PLA-MA600 and PLA-MA1200 in Table 1, respectively.

### 2.2. Preparation of Solutions

In the presence of water, the oligolactide chains hydrolyze to form lactic acid, which leads to the acidification of the environment. Correspondingly, we observed this experimentally in deionized water, where the acidity of solutions of graft copolymers P(NIPAM-*g*-PLA) decreased to pH = 4–5 within 48 h. Since nitroxyl radicals transform to their diamagnetic form in acidic media [30], the phosphate-buffered solution (PBS, pH = 7.4) was used as an aqueous medium. The buffer capacity of the solution was sufficient to maintain the neutral pH of the medium for a month. The pH of the prepared PBS was monitored using a pre-calibrated pH meter/millivoltmeter. Predefined amounts of **II**, **III** and **IV** were dissolved in a ~0.5 mM TEMPO solution in PBS to make 5 wt% and 10 wt% polymer solutions. Due to the inconvenience of working with highly viscous solutions, only 5 wt% solutions of **V** were prepared. The dissolution of the thermoresponsive graft copolymers was carried out across 24 h at 277 K. Afterwards, ~5–6 mg of the resulting solutions was placed into glass capillary tubes with a 2 mm inner diameter and sealed to prevent evaporation.

### 2.3. EPR Spectroscopy

EPR spectra were recorded using the X-band Bruker EMX-500 spectrometer (Bruker, Karlsruhe, Germany). The temperature of the samples was set in the 273–363 K range using the flow of nitrogen gas. The thermostatic device of Bruker was used; the accuracy of temperature setting was about ±0.5 K. Each sample was kept at its predefined temperature for 5 min to equilibrate. Typical parameters of spectra recording were 1 mW microwave power, modulation amplitude of 0.04 mT and 8 mT sweep width. The suppression of fast spin probes EPR signal in the PBS phase was performed by adding 10 mg of CuCl_2_∙2H_2_O to 0.5 mL of polymer solutions, as recommended in [26].

### 2.4. EPR Spectra Treatment and Simulation

The amplitudes and linewidths of the spectral lines were measured using the EsrD program (Chemistry Department of Lomonosov Moscow State University) [31]. All spectra simulations were performed using homemade scripts for the MATLAB program, employing an Easyspin (v. 5.2.28) toolbox [32]. Spectra of TEMPO radicals in solutions and globules were simulated as a ‘chili’ function in Easyspin. The slow-motion ‘chili’ model is based on the Schneider–Freed theory [33], solving equations for slow-tumbling nitroxides. The anisotropic values of spin Hamiltonian parameters (g-tensor and hyperfine coupling (hfc) tensor, usually referred to as “a-tensor”) were averaged to obtain g_iso_ and a_iso_ values. Rotational correlation time tensor (t_corr_) was calculated from the averaged rotational diffusion constant. Further details of spectra simulation are presented in Appendix A.

## 3. Results

**Figure 2:**
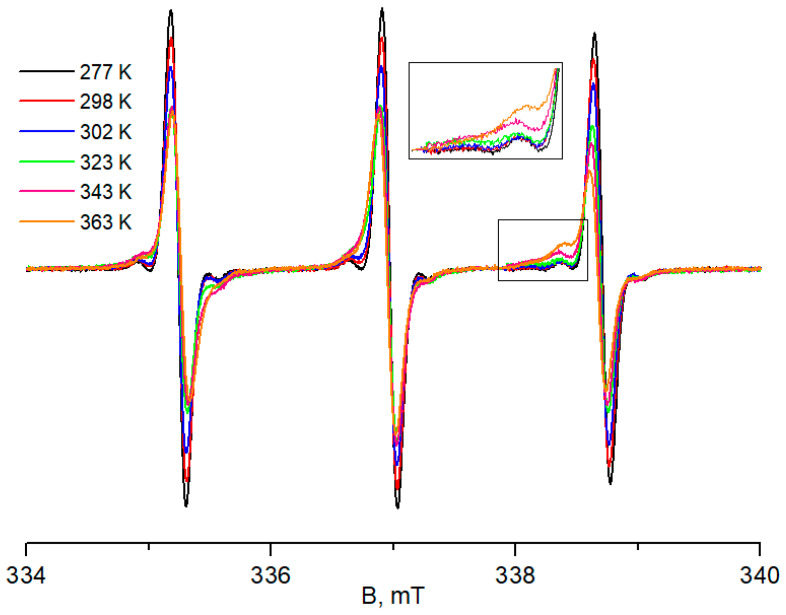
EPR spectra of TEMPO in polymer **IV** solutions (5 wt%, PBS) at 277–363 K.

The line shapes of TEMPO spectra in all studied polymer solutions at 273–277 K appear similar to those in aqueous solutions and constitute narrow triplets arising from the hyperfine interaction between the unpaired electron and the paramagnetic ^14^N nuclei with nuclear spin I = 1. Such shapes are typical for the spectra of fast-rotating nitroxides (denoted as species of type **A**) (t_corr_ < 10 ps), also known as fast-motion limit spectra [34] (Figure 2). Fast rotation leads to the averaging of paramagnetic interactions in different orientations and manifests in three equal linewidth and amplitude symmetric lines. In addition to hyperfine splittings, low-intense satellites are observed as additional splittings for each of the three lines. These satellites arise due to hyperfine interactions with the paramagnetic ^13^C nuclei (I = ½) in the methyl groups, as well as the carbon atoms in the piperidine ring. These spectra were successfully simulated in accordance with the isotropic rotation model. The isotropic values of hyperfine coupling (hfc) constants a_iso_ and rotational correlation times t_corr,iso_ obtained from the simulation are presented in Table 2. The values of a_iso_ for TEMPO in all solutions below VPTT are almost independent of the polymer composition, pointing to similar environments for the spin probe in pure PBS and in the solutions of polymers. According to the values of rotational correlation times (see Table 2), the rotation of TEMPO molecules in 5 wt% and 10 wt% polymer solutions with the highest oligolactide content (samples **II**–**V**) is restrained compared to pure PBS and PNIPAM solutions. This indicates higher viscosity for the solutions of polymers **II**–**V**.

In the solutions of TEMPO in PBS, the slight broadening of the EPR spectrum is observed when temperature increases in the 273–353 K range. This broadening becomes noticeable above 303 K. Apparently, it is mostly related to spin exchange broadening [35]. The a_iso_ hyperfine splitting constant decreases with heating, manifesting in the decrease in the distance between the extreme components of the triplet spectrum. The a_iso_ changes occur due to the decrease in water dielectric permittivity upon heating [36].

Figure 2 depicts the changes in the EPR spectra of the TEMPO radical in a 5 wt% solution of polymer **IV**, exemplifying the spectral changes in the studied solutions of copolymers. The intensity of the signal in the TEMPO spectra in 5 wt% solution of polymer **IV** slightly diminishes at 277–298 K due to the broadening of the spectral lines, similarly to the TEMPO signal in the PBS. Starting at 298 K (denoted as T_start_, see Table 2), the amplitude of the signal starts to drop down faster, while the number of paramagnetic species remains constant. Initially, this is only related to signal broadening. However, at 333–343 K, an additional component appears due to the splitting of the high-field line. Similar trends are observed for 5 wt% and 10 wt% solutions of other studied polymers.

Such changes in the EPR spectra of TEMPO in the solutions of thermoresponsive polymers were previously observed for PNIPAM [26] and poloxamer copolymers [27] in aqueous solutions. It was suggested that the broadened, and hence less intensive, signal appearing at temperatures above VPTT belonged to TEMPO radicals located in less polar and denser media, namely, polymer globules representing the initial step of coil-to-globule transition. The main difference between homo- and copolymers is that the transition in PNIPAM solutions occurs in a much narrower temperature range.

The simulation of the EPR spectra can provide data corresponding to the ratios of the number of paramagnetic species in solution vs. globules, as well as to the properties of the globules (local polarity, microviscosity, etc.). However, modelling the experimental spectra as sums of the spectra for two kinds of species—**A**, TEMPO in aqueous solution, and **B**, TEMPO in polymer globules—are ambiguous due to their superposition. A way to solve this problem is to “kill” the signal of the rapidly rotating radicals **A** in aqueous solution due to spin exchange broadening in the EPR spectra in the presence of inert paramagnetic particles, e.g., Cu^2+^ ions. As shown earlier [26], the optimal concentration of Cu^2+^ ions is about 0.2 M. Experimentally, the addition of 10 mg of CuCl_2_∙2H_2_O to 0.5 mL of the solutions of polymers **II**–**V** at temperatures below 285 K results in the spectra of fast-motion type **A** species merging with the baseline. Further heating in the presence of Cu^2+^ ions leads to a signal arising with its shape significantly different from that in the solutions. Similar changes were also observed for homopolymer **I** [26]. They can be attributed to the spin probes located in the polymer globules (type **B** species).

The temperatures of initial appearance for the signal (T_glob_) in different polymer solutions are given in Table 3. No significant difference between T_glob_ for 5 and 10 wt% solutions is observed. Increasing oligolactide content in the copolymers decreases the T_glob_ values. For instance, T_glob_ drops from 302 K for the homopolymer **I** solution to 286 K for the solution of copolymer **V**. In all cases the T_glob_ values are lower than the values for volume phase transition temperature (VPTT), measured via macroscopic methods. For example, in polymer **V** solutions, the globules initially appear at temperatures that are 15 degrees lower than VPTT measured via transmission. Similar formation of polymer aggregates in graft copolymers P(NIPAM-*g*-PLA) was also observed previously by dynamic light scattering (DLS) by Ksendzov et al. [5]. Aggregates of 17–23 nm could be already detected at 283 K in 0.25 wt% of **II** and **III** aqueous solutions, with the formation of bigger aggregates (50–75 nm) taking place above 303 K.

**Figure 3:**
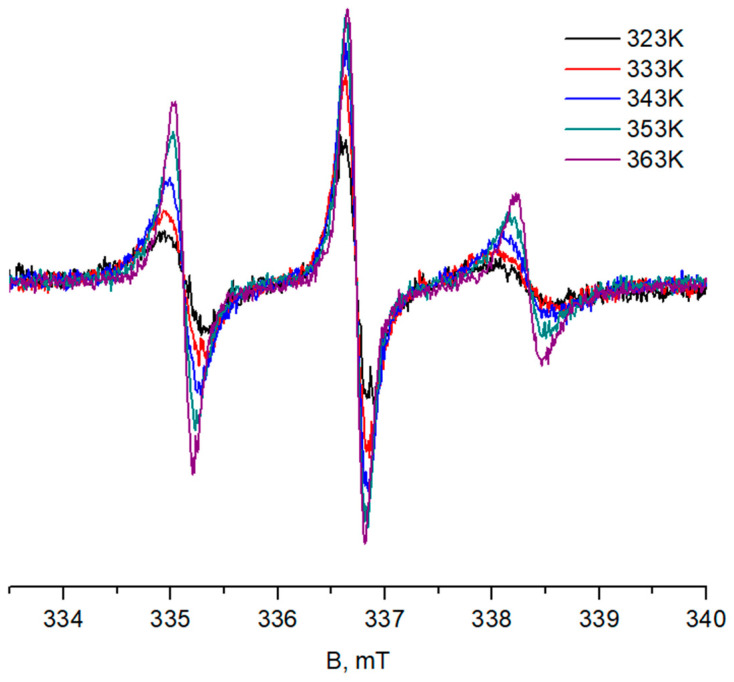
Spectra of TEMPO in globules in copolymer **V** solution (5 wt%, PBS) recorded in the presence of Cu^2+^ ions at 323–363 K.

At 276–323 K, the line shapes of the TEMPO spectra in the presence of Cu^2+^ ions do not change significantly for all the studied copolymers. As the temperature rises, the volume of the globules increases, resulting in increases for the spectral intensity (the corresponding data for copolymer **V** solution is presented in Figure 3). Above 323 K, line narrowing occurs, with the high-field component of TEMPO in all studied P(NIPAM-*g*-PLA) solutions undergoing a high-field shift at 333 K in the presence of Cu^2+^ ions.

The narrowing of the spectra above 323 K apparently corresponds to the increase in TEMPO mobility in the globules associated with the increase in the polymer chains mobility in 5 wt% and 10 wt% solutions. In addition, oligolactide chains may swell in water at high temperatures, also influencing spin probe mobility. The shift of the high-field component may occur due to the probe exchange between the globules and the external solution. In other words, the emerging inhomogeneities may be dynamic in nature.

**Figure 4:**
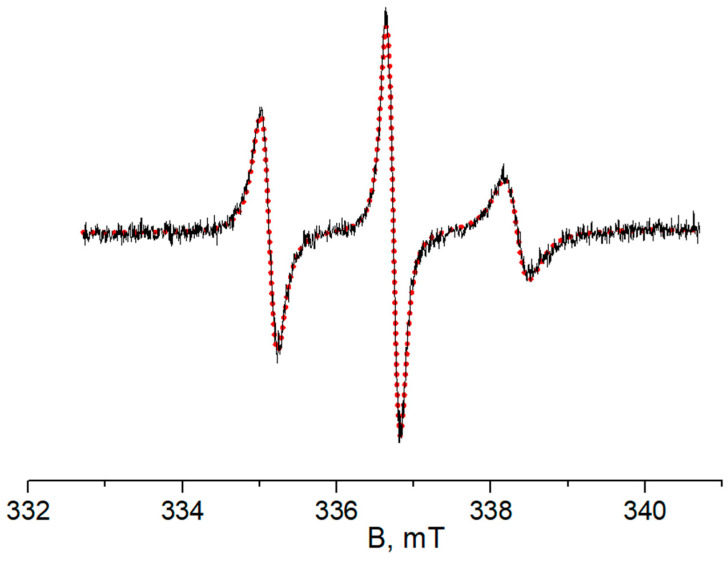
EPR spectra of TEMPO in globules of polymer **V** solution (5 wt%, PBS) in the presence of Cu^2+^ ions recorded at 353 K. Black line—experimental spectrum, red dots—simulated spectrum. Parameters of simulated spectrum: g_iso_ = 2.00607, a_iso_ = 1.60 mT, t_corr, x_ = 16 ns, t_corr, y_ = 0.3 ns, t_corr, z_ = 1.7 ns (t_corr,iso_ =0.7 ns).

The EPR spectra of TEMPO in 5 and 10 wt% P(NIPAM-*g*-PLA) solutions in the presence of Cu^2+^ ions (type **B** species) at different temperatures were simulated within the slow-motion model with anisotropic rotation of the nitroxide radical mostly hindered along the X axis. An example of such simulation is presented in Figure 4. Selected paramagnetic and dynamic parameters of the experimental EPR spectra at 343 K are presented in Table 4. Full simulation results, including hfc constants, g-factors and rotational correlation times, are given in Appendix A.

Isotropic hyperfine coupling constants (a_iso_) of TEMPO in the globules of copolymers **II**–**IV** at 343 K are lower than those of TEMPO in the PBS solution and are similar to those in the homopolymer **I** solution and in the individual TEMPO solution in nitromethane [37]. Notably, the polarity of polymer **V** globules is lower and similar to that in dimethylformamide [37]. The values of spin Hamiltonian (a_iso_, g_iso_) and dynamic (rotational correlation time t_corr_) parameters of TEMPO in globules of **III** and **IV** do not depend on the concentration of the polymer and molecular mass of the oligolactide. This points to the rigidity and the polarity of the globules being independent of polymer concentration (at 5–10 wt%) and molar mass of oligolactide (600–1200 Da).

As the temperature increases from 343 K to 363 K, the a_iso_ values of particles **B** in the globules of copolymers **III**–**V** rises from 1.58–1.60 mT to 1.61–1.62 mT. The increase in a_iso_ may be associated with the probe molecule exchange between the polar external solution and the hydrophobic globules. Since the a_iso_ values in the external aqueous solution are higher than in the globules, the exchange leads to an increase in the measured value of a_iso_ for species **B**. A similar exchange was observed in the aqueous solutions of polyoxamers copolymers [27], indicating the presence of dynamic inhomogeneities in solutions of copolymers.

**Figure 5:**
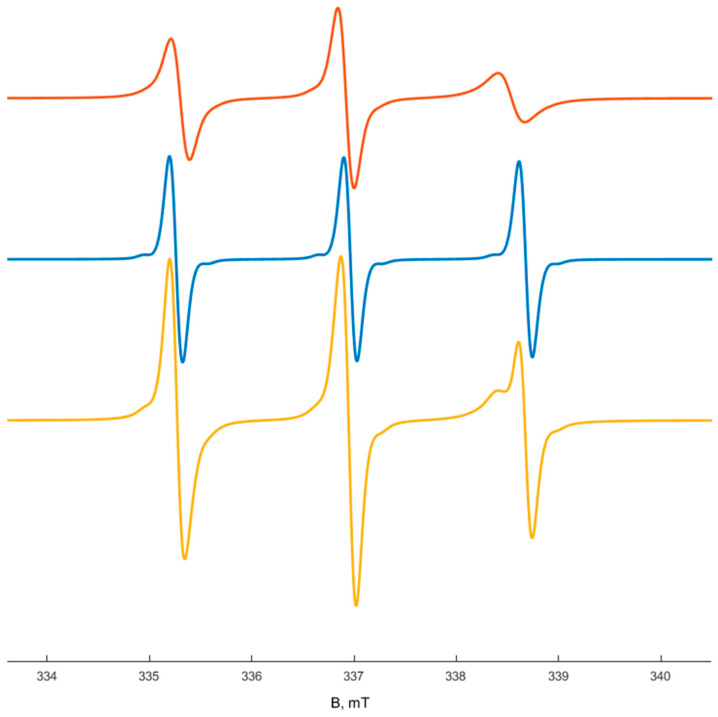
Deconvolution for the EPR spectra of TEMPO in the polymer **IV** solution (10 wt%): TEMPO in globules (red line), TEMPO in external solution (blue line), experimental spectrum of TEMPO in globules and external solution (yellow line).

The spin Hamiltonian and dynamic parameters of TEMPO evaluated from spectra recorded in the presence of Cu^2+^ ions were used as initial for type **B** particles when simulating the TEMPO spectra in P(NIPAM-*g*-PLA) solutions. The parameters for type **A** species in the external aqueous solution were taken from the simulation of spectra of TEMPO in the PBS solution (see Appendix A, Table A1, Table A2 and Table A3). An example for the deconvolution of the EPR spectrum of TEMPO in solutions of graft copolymers P(NIPAM-*g*-PLA) into the individual spectra of species A and B is presented in Figure 5.

When heating from 295 K to 363 K, the a_iso_ value for type **A** TEMPO radicals in P(NIPAM-*g*-PLA) solutions falls from 1.73 mT to 1.71 mT, indicating a decrease in water polarity. These changes are similar to those reported for TEMPO in PBS. The paramagnetic and dynamic parameters of type **B** species also change with temperature. The a_iso_ value is a constant up to 323 K, increasing from 1.58 mT to 1.62 mT at 363 K. This points to the formation of dynamic inhomogeneities at temperatures as low as 323 K. In contrast, the hyperfine splitting constant of type **B** species in PNIPAM solutions does not change in the range of 305–353 K, supporting the formation of static inhomogeneities. The temperature dependence of average rotational correlation time t_corr,iso_ of type **B** probes in copolymer solutions shows that for all copolymers, t_corr,iso_ reaches T_max_ values, in the range of 308–313 K and 306 K for copolymers **II**–**IV** and for polymer **V**, respectively. A similar trend was observed for PNIPAM solutions; however, T_max_ was significantly higher (323 K).

**Figure 6:**
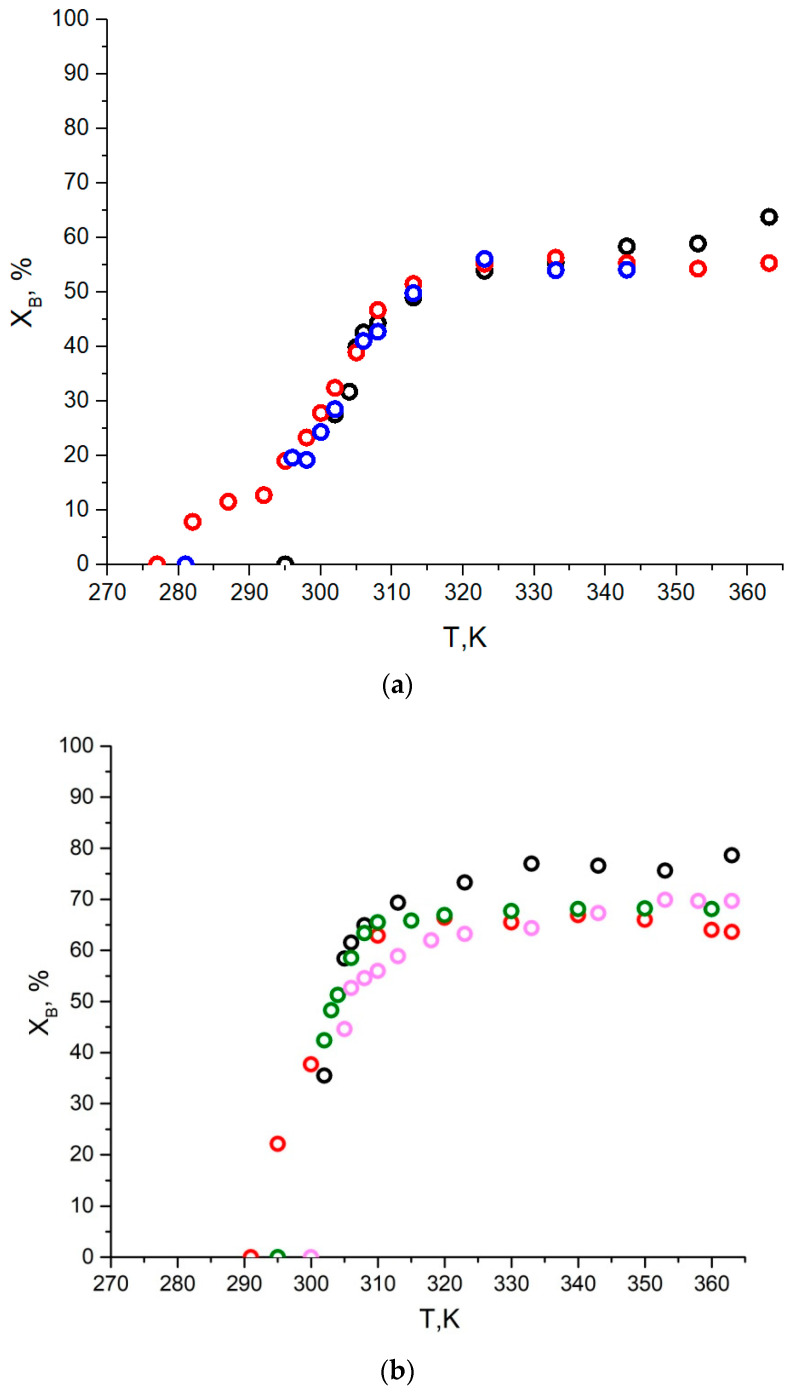
Temperature dependence of probe quantity in globules (X_B_) measured as a result of EPR spectra modeling (**a**) for 5 wt% solutions, (**b**) for 10 wt% solutions. (**I**—magenta, **II**—green, **III**—black, **IV**—red dots, **V**—blue dots). The data for homopolymer **II** was taken from [26].

Besides the paramagnetic and dynamic parameters of the spin probes in the globules, the changes in TEMPO content in the globules vs. temperature were estimated from the simulations of the EPR spectra. Apparently, the amount of type **B** probes increases smoothly with an inflection point near VPTT (see Figure 6). Above 323 K, the content of type **B** species is about 55–60% and 65–70% for 5 wt% and 10 wt% solutions, respectively. Additionally, for the 5 wt% PBS solution (see Figure 6a), the changes in TEMPO content with heating are smoother than those for the 10 wt% solutions for the same copolymer. Hence, for copolymer **IV**, the temperature range of TEMPO content increasing up to the stationary concentration is broader for the 5 wt% solutions (about 35 degrees), as compared to the 10 wt% solutions (about 20 degrees). Moreover, the TEMPO content in the copolymer globules increases more smoothly when passing the inflection points (see Figure 6b), in contrast to the PNIPAM solutions, where the concentration of type **B** species rises steeply with a 40% increase at LCST (305 K).

## 4. Discussion

According to the spin probe EPR data, the coil-to-globule transition in the aqueous solutions of graft copolymers of PNIPAM with oligolactide proceeds gradually in a broad temperature range (about 20–35 degrees) where the first polymer aggregates form below VPTT, confirmed via turbidimetry or DSC. These results agree with the data measured for P(NIPAM-*g*-PLA) solutions via dynamic light scattering. However, the spin probe approach in EPR spectroscopy not only allowed us to confirm the existence of nanoscopic inhomogeneities at temperatures lower than VPTT but also to investigate the nature and properties of these inhomogeneities, including their polarity and rigidity. According to the a_iso_ values of the spin probe in the globules at 300–315 K measured in the simulated EPR spectra, the collapsed globules of P(NIPAM-*g*-PLA) are more hydrophobic than those of PNIPAM. The hydrophobicity of the polymer surface usually determines the adhesion in mammalian cells [38], as well as the adsorption of proteins [39] of extracellular matrices, such as collagen and fibronectin. Hence, we assume that the surface of graft copolymers will be more adhesive for cells and proteins.

The inhomogeneities in the studied solutions of graft copolymers formed via collapsing polymer chains become dynamic once heated above VPTT. In other words, tracer molecules are exchanged between the hydrophobic (mostly polymeric aggregates) and the hydrophilic (external aqueous solution) media. This is supported by the increase in a_iso_, the measured hyperfine coupling constant of the probes in the globules, to less “hydrophobic” values, as well as by the increase in probe mobility in the polymeric globules. An increase in grafting density results in the transformation of static inhomogeneities into dynamic ones at lower temperatures. These temperatures may be regarded as the onset points of the increase in a_iso_ values, with the temperature values dropping with the increase in grafting density. An increase in probe mobility related to the mobility of the polymer chains and manifested itself as a decrease in rotational correlation time for the spin probes in the globules, which also indicates the possibility of probe exchange between the hydrophobic and hydrophilic environments. Apparently, the oligolactide groups in the graft copolymers make the collapsed globules looser with a higher free volume, in comparison with PNIPAM, adding as little as 3% of oligolactide results in the formation of dynamic inhomogeneities. The increase in oligolactide content expands the temperature range, corresponding to the existence of these inhomogeneities. The grafting density of oligolactide not only influences the isotropic mobility of the probe in the globule but also affects the preferential directions of its movement. According to the data in Table 4, the anisotropy of the tensor of rotational diffusion diminishes with the increase in oligolactide content.

The differences in temperature dependences of TEMPO content in the polymer globules between 5 and 10 wt% solutions indicate smoother coil-to-globule phase transition for lower polymer concentrations (see Figure 6). Apparently, polymer–polymer interactions in the 10 wt% solutions below VPTT are stronger than in the 5 wt% solutions, and these interactions may lead to a fast and sharp collapse for the globule [40]. In contrast, the interactions between water molecules and polymer chains may prevail for the 5 wt% solutions, with more steps required for hydrogen bond dissociation. Consequently, the globule collapse occurs in a wider temperature range for more dilute solutions. Previously, similar concentration dependences for the temperature ranges of coil-to-globule transitions were measured for the 1–5 wt% PNIPAM aqueous solutions [41].

The observed peculiarities of coil-to-globule transitions in the aqueous solutions of graft P(NIPAM-*g*-PLA) should be accounted for in the biomedical applications of these copolymers. The inhomogeneities being already present in this system at 285 K together with the broadened phase transition may result in some difficulties during the detachment of cell sheets from the coatings based on graft P(NIPAM-*g*-PLA) copolymers. The polymer chains and polymer aggregates may penetrate the extracellular matrix and inhibit the process of detachment while cooling. On the other hand, the existence of dynamic inhomogeneities at physiological temperatures (310 K) expands the range of possible applications for the studied polymers. The tracer molecule exchange between the globules and the solutions allows for the application of graft P(NIPAM-*g*-PLA) in tissue regeneration as injectable drug-containing gels. The bioactive compounds captured by the collapsed polymeric globules in the polymer gel could potentially be released in vivo due to the exchange with the external solutions.

## 5. Conclusions

The spin probe technique of CW EPR spectroscopy was used to investigate coil-to-globule transitions in the aqueous solutions of thermoresponsive graft copolymers of NIPAM with biodegradable oligolactide chains upon heating from 276 K to 363 K. According to the measured paramagnetic parameters of the spin probe (TEMPO) in the globules, graft copolymer aggregates have lower polarity than those of PNIPAM. The presence of dynamic inhomogeneities, allowing for the probe molecule exchange between the hydrophobic and hydrophilic regions was observed at temperatures above T_max_. With the graft density increasing, the P(NIPAM-*g*-PLA) globules become looser, with the probe molecule exchange initiating at lower temperatures. The discovered peculiarities expand the possibilities for the biomedical application of P(NIPAM-*g*-PLA) in tissue engineering as injectable hydrogels.

## Figures and Tables

**Table 1:** Characteristics of the polymers and the abbreviation used [5,26]. Mn is the number average molecular weight, Ð is polydispersity.

No.	Polymer	Mn, kDa	Đ	VPTT (DSC/Turbidimetry), K
**I**	PNIPAM	107.6	2.03	305/305
**II**	P(NIPAM-*g*-PLA-MA600) 97:3	23.5	1.25	304/299
**III**	P(NIPAM-*g*-PLA-MA1200) 97:3	25.7	1.23	305/300
**IV**	P(NIPAM-*g*-PLA-MA600) 91:9	27.7	1.23	301/295
**V**	P(NIPAM-*g*-PLA-MA1200) 83:17	30.8	1.23	302/300

**Table 2:** Hfc constants a_iso_ and rotational correlation time t_corr,iso_ for TEMPO in solutions of thermoresponsive polymers and VPTT of the polymers in 5 wt% and 10 wt% aqueous solutions. T_sim_—the temperature of solutions, the spectra of which were simulated. T_start_ —the initial temperature for the decay of the signal amplitude.

System, Concentration	T_sim_, K	a_iso_, mT	t_corr,iso_, ps	T_start_, K	VPTT (DSC/Turbidimetry), K
TEMPO/PBS	273	1.74	12	-	-/-
**I**, 10 wt% ^1^	295	1.73	11	305 ± 1	305/305
**III**, 5 wt%	281	1.74	22	301 ± 2	305/300
**IV**, 5 wt%	277	1.74	12	298 ± 3	301/-
**V**, 5 wt%	276	1.74	29	298 ± 2	302/300
**II**, 10 wt%	295	1.73	30	299 ± 1	-
**III**, 10 wt%	273	1.74	30	298 ± 1	-
**IV**, 10 wt%	276	1.74	50	298 ± 1	-

^1^ Data from [26].

**Table 3:** Onset temperatures for globule formation in the solutions of P(NIPAM-*g*-PLA) in the presence of Cu^2+^ ions measured via EPR.

System	Concentration	T_glob_, K
**I**	10 wt%	302
**III**	5 wt%	298
**III**	10 wt%	296
**IV**	5 wt%	293
**IV**	10 wt%	294
**V**	5 wt%	286

**Table 4:** A_iso,_ components of rotational correlation time tensors and isotropic rotational correlation time of TEMPO (type **B** species) in P(NIPAM-*g*-PLA) solutions in the presence of Cu^2+^ ions at 343 K.

Polymer	Concentration	a_iso_, mT	t_corr,x_, ns	t_corr,y_, ns	t_corr,z_, ns	t_corr,iso_, ns
**I** ^1,2^	10 wt%	1.60	25	0.3	1.9	0.9
**II** ^1^	10 wt%	1.59	25	0.4	1.9	1.1
**III**	10 wt%	1.60	25	0.4	2.0	0.9
**III**	5 wt%	1.60	25	0.4	2.1	1.1
**IV**	10 wt%	1.60	19	0.4	2.2	1.1
**IV**	5 wt%	1.60	19	0.4	2.2	1.1
**V**	5 wt%	1.58	17	0.4	2.3	0.9

^1^ Data from modeling the spectra of TEMPO polymer solutions without Cu^2+^ ions. ^2^ [26].

## Data Availability

Not applicable.

## Appendix A. EPR Spectra Simulation Details

All CW EPR spectra simulations were performed with the MATLAB program package employing the Easyspin (v. 5.2.28) toolbox [32]. The quality of fitting was controlled by the calculation of root-mean-square deviation for the difference spectra. The root-mean-square deviation obtained from simulations of all fitted spectra was less than 1%. Spectral and dynamic parameters were obtained by fitting the simulated EPR spectra to experimental data using least-squares fitting algorithms [42].

The simulations of the spectra of radicals in PBS and polymer solutions as spectra of type A probes were carried out in an isotropic rotation model implemented as a ‘garlic’ function in Easyspin. Line broadening was defined in relation to isotropic rotation correlation time, as well as via additional convolution of Gaussian and Lorentz line broadening. In addition to the splittings on the ^14^N nuclei for type A probes, isotropic hyperfine splittings on the ^13^C isotopes in six CH_3_-groups with a_iso_ = 0.54 mT were accounted for. To simplify the modeling procedure, we changed the abundances for carbon isotopes instead of adding six paramagnetic nuclei. The following initial diagonal values of g-tensor and hyperfine interaction a_N_-tensor were used:

g_x_ = 2.0092, g_y_ = 2.0062, g_z_ = 2.0023;

A_xx_ = 0.73 mT, A_yy_ = 0.73 mT, A_zz_ = 3.65 mT.

The simulations of the spectra of radicals in polymer globules (type B) were carried out in a slow-motion mode with the ‘chili’ function of Easyspin. The slow-motion ‘chili’ model is based on the Schneider–Freed theory [33], solving equations for slow-tumbling nitroxides. Line broadening was defined in relation to the anisotropic rotation correlation time and additional Gaussian line broadening. The following initial diagonal values of g-tensor and hyperfine interaction a-tensor were used:

g_x_ = 2.0098, g_y_ = 2.0061, g_z_ = 2.0023;

A_xx_ = 0.73 mT, A_yy_ = 0.73 mT, A_zz_ = 3.35 mT.

The initial values of magnetic parameters were taken from [43]. In the simulation, only the g_x_ and A_zz_ components were being varied, as they are more sensitive to the environment. The isotropic g_iso_ and a_iso_ values were calculated as averages of the diagonal elements:

$$g_{\mathrm{iso}}=\frac{1}{3}\left(g_{x}+g_{y}+g_{z}\right)$$

$$a_{\mathrm{iso}}=\frac{1}{3}\left(A_{\mathrm{xx}}+A_{\mathrm{yy}}+A_{\mathrm{zz}}\right)$$

The rotational correlation times along the different axes and the line widths could only be varied independently due to their simultaneous influence on the line shape. The averaged (isotropic) correlation time t_corr,iso_ was calculated from isotropic rotational diffusion constant using the following equations:

$$D_{rot}=\frac{1}{3}\left(D_{xx}+D_{yy}+D_{zz}\right)$$

$$D_{rot}=\frac{1}{6t}$$

The spectra of TEMPO radicals in solutions (type A + type B) were simulated using the ‘chili’ function in Easyspin. The initial parameters for type A probes were taken from the spectra simulations of the TEMPO radical in PBS at different temperatures. The initial parameters for type B probes were taken from the spectra simulations of the TEMPO radical in the polymer solutions recorded in the presence of Cu^2+^ ions at 343–363 K. χ_B_ was calculated from weight values of type B particles in the full EPR spectra, assuming that the weight of type A particles was always equal to 1.

$$\chi_{B}=\frac{\mathrm{weight}\left(B\right)}{1+\mathrm{weight}\left(B\right)}\cdot 100\%$$

The uncertainties for simulated parameters were calculated, as recommended in [44] and they were estimated as follows:

a_iso_ ± 0.01 mT

g_iso_ ± 0.00003

t_corr,x_ ± 3 ns

t_corr,y_ ± 0.1 ns

t_corr,z_ ± 0.2 ns

**Table A1:** Selected spectral parameters from simulation TEMPO EPR spectra in 5 and 10 wt% solutions in the presence of a quencher at 343–363 K.

Polymer, Concentration	T, K	g_iso_	a_iso_, mT	t_corr,x_, ns	t_corr,y_, ns	t_corr,z_, ns	t_corr,iso_, ns
**I**, 10 wt%	353	2.00615	1.60	21.9	1.9	0.3	0.7
**III**, 10 wt%	363	2.00620	1.62	17.8	0.2	1.0	0.6
	353	2.00620	1.60	25.1	0.3	1.8	0.7
	343	2.00617	1.60	25.1	0.4	2.0	0.9
**III**, 5 wt%	363	2.00610	1.62	17.8	0.3	1.1	0.7
	353	2.00607	1.60	25.1	0.3	1.8	0.8
	343	2.00607	1.60	25.1	0.4	2.1	1.1
**IV**, 10 wt%	363	2.00613	1.62	17.8	0.3	1.1	0.7
	353	2.00613	1.60	17.8	0.3	1.9	0.8
	343	2.00607	1.60	18.6	0.4	2.2	1.1
**IV**, 5 wt%	363	2.00597	1.62	17.8	0.3	1.1	0.7
	353	2.00597	1.60	17.8	0.3	1.9	0.8
	343	2.00590	1.60	18.6	0.4	2.2	1.1
**V**, 5 wt%	363	2.00610	1.61	9.8	0.2	1.0	0.5
	353	2.00607	1.60	16.2	0.3	1.7	0.7
	343	2.00607	1.58	16.6	0.4	2.3	0.9

**Table A2:** Selected spectral parameters from simulation TEMPO EPR spectra in 5 and 10 wt% solutions at 295–363 K.

**II, 10 wt% solution**							
**T, K**	**Type A**			**Type B**			
	**g** **_iso_**	**a_iso_, mT**	**t_corr,iso_, ps**	**g** **_iso_**	**a_iso_, mT**	**t_corr,iso_, ns**	**X_B_, %**
360	2.00591	1.72	10.0	2.00609	1.62	0.7	68.1
350	2.00590	1.72	10.0	2.00611	1.60	0.8	68.2
340	2.0059	1.72	10.0	2.00606	1.59	1.1	68.1
330	2.00588	1.73	10.0	2.00602	1.58	1.3	67.7
320	2.00586	1.73	10.0	2.00598	1.57	1.7	66.9
315	2.00586	1.73	10.0	2.00595	1.57	1.8	65.8
310	2.00585	1.73	10.0	2.00588	1.57	1.9	65.5
308	2.00585	1.73	10.0	2.00589	1.57	1.9	63.4
306	2.00585	1.73	10.0	2.00588	1.57	1.9	58.5
304	2.00586	1.73	10.0	2.00587	1.57	1.8	51.3
303	2.00584	1.73	10.0	2.00587	1.57	1.8	48.3
302	2.00585	1.73	10.0	2.00587	1.57	1.7	42.4
360	2.00591	1.72	10.0	2.00609	1.62	0.9	68.1
350	2.00590	1.72	10.0	2.00611	1.60	0.8	68.2
**III, 10 wt% solution**							
**T, K**	**Type A**			**Type B**			
	**g** **_iso_**	**a_iso_, mT**	**t_corr,iso_, ps**	**g** **_iso_**	**a_iso_, mT**	**t_corr,iso_, ns**	**X_B_, %**
363	2.00591	1.71	10.0	2.00609	1.62	0.6	78.6
353	2.00590	1.72	10.0	2.00608	1.60	0.8	75.6
343	2.00590	1.72	10.0	2.00605	1.60	1.0	76.6
333	2.00589	1.72	10.0	2.00602	1.59	1.2	77.0
323	2.00587	1.73	10.0	2.00598	1.58	1.5	73.3
313	2.00586	1.73	10.0	2.00596	1.57	1.9	69.3
308	2.00585	1.73	10.0	2.00588	1.57	1.8	64.9
306	2.00585	1.73	10.0	2.00586	1.57	1.8	61.5
305	2.00585	1.73	10.0	2.00584	1.57	1.8	58.4
304	2.00583	1.73	10.0	2.00581	1.57	1.6	51.3
302	2.00580	1.73	10.0	2.00570	1.57	1.6	35.5
**III, 5 wt% solution**							
**T, K**	**Type A**			**Type B**			
	**g** **_iso_**	**a_iso_, mT**	**t_corr,iso_, ps**	**g** **_iso_**	**a_iso_, mT**	**t_corr,iso_, ns**	**X_B_, %**
363	2.00584	1.71	10.0	2.00602	1.62	0.6	63.8
353	2.00583	1.72	10.0	2.00601	1.60	0.8	58.9
343	2.00582	1.72	10.0	2.00596	1.60	1.0	58.4
333	2.00581	1.72	10.0	2.00591	1.59	1.3	55.4
323	2.00580	1.73	10.0	2.00585	1.58	1.6	53.9
313	2.00580	1.73	10.0	2.00580	1.56	2.0	49.0
308	2.00580	1.73	10.0	2.00574	1.56	2.0	44.3
306	2.00579	1.73	10.0	2.00574	1.56	2.0	42.6
305	2.00579	1.73	10.0	2.00574	1.56	2.0	39.9
304	2.00579	1.73	10.0	2.00574	1.56	2.0	31.7
302	2.00579	1.73	10.0	2.00574	1.56	2.0	27.5
**IV, 10 wt% solution**							
**T, K**	**Type A**			**Type B**			
	**g** **_iso_**	**a_iso_, mT**	**t_corr,iso_, ps**	**g** **_iso_**	**a_iso_, mT**	**t_corr,iso_, ns**	**X_B_, %**
363	2.00586	1.71	10.0	2.00608	1.62	0.6	63.6
360	2.00586	1.71	10.0	2.00607	1.62	0.6	64.0
350	2.00585	1.72	10.0	2.00607	1.60	0.8	66.0
340	2.00585	1.72	10.0	2.00602	1.59	1.0	66.9
330	2.00584	1.72	10.0	2.00601	1.59	1.3	65.5
320	2.00584	1.73	10.0	2.00596	1.58	1.6	66.4
310	2.00584	1.73	10.0	2.00594	1.58	1.9	62.9
300	2.00584	1.73	12.4	2.00584	1.58	1.9	37.7
295	2.00585	1.73	18.7	2.00577	1.58	1.9	22.1
**IV, 5 wt% solution**							
**T, K**	**Type A**			**Type B**			
	**g** **_iso_**	**a_iso_, mT**	**t_corr,iso_, ps**	**g** **_iso_**	**a_iso_, mT**	**t_corr,iso_, ns**	**X_B_, %**
363	2.00594	1.71	10.0	2.00613	1.62	0.7	55.3
353	2.00593	1.72	10.0	2.00613	1.60	0.8	54.3
343	2.00592	1.72	10.0	2.00607	1.60	1.0	55.3
333	2.00591	1.72	10.0	2.00603	1.58	1.3	56.3
323	2.00590	1.73	10.0	2.00600	1.56	1.7	55.2
313	2.00589	1.73	10.0	2.00595	1.56	2.0	51.5
308	2.00590	1.73	10.0	2.00592	1.56	2.1	46.7
305	2.00589	1.73	10.0	2.00588	1.56	2.1	38.9
302	2.00591	1.73	10.0	2.00587	1.56	2.0	32.4
300	2.00591	1.73	11.0	2.00587	1.56	2.0	27.8
298	2.00588	1.73	12.2	2.00587	1.56	2.0	23.3
295	2.00593	1.73	12.2	2.00587	1.56	2.0	19.0
292	2.00590	1.73	12.2	2.00587	1.56	0.5	12.7
287	2.00586	1.73	12.2	2.00587	1.56	0.8	11.5
282	2.00590	1.74	12.2	2.00587	1.56	0.2	7.8
**V, 5 wt% solution**							
**T, K**	**Type A**			**Type B**			
	**g** **_iso_**	**a_iso_, mT**	**t_corr,iso_, ps**	**g** **_iso_**	**a_iso_, mT**	**t_corr,iso_, ns**	**X_B_, %**
343	2.00585	1.72	10.0	2.00603	1.58	1.0	54.1
333	2.00585	1.72	10.0	2.00595	1.58	1.4	54.0
323	2.00583	1.73	10.0	2.00587	1.56	1.9	56.1
313	2.00577	1.73	10.0	2.00587	1.56	1.9	49.8
308	2.00576	1.73	10.0	2.00587	1.56	1.9	42.7
306	2.00576	1.73	10.0	2.00587	1.56	2.2	41.0
302	2.00575	1.73	10.0	2.00587	1.56	0.8	28.5
300	2.00575	1.73	11.3	2.00587	1.56	0.8	24.3
298	2.00575	1.73	11.3	2.00587	1.56	0.8	19.2
296	2.00576	1.73	11.3	2.00587	1.56	1.2	19.6

**Table A3:** Selected spectral parameters from simulation TEMPO EPR spectra in PBS at 273–353 K.

**T, K**	**a_iso_, mT**	**t_corr,iso_, ps**
273	1.74	12.1
283	1.74	10.9
293	1.73	10.0
303	1.73	10.0
313	1.73	10.0
323	1.73	10.0
333	1.72	10.0
343	1.72	10.0
353	1.72	10.0

## References

[1] Ward M.A., Georgiou T.K. (2011). Thermoresponsive Polymers for Biomedical Applications. Polymers.

[2] Roy D., Brooks W.L.A., Sumerlin B.S. (2013). New Directions in Thermoresponsive Polymers. Chem. Soc. Rev..

[3] Zhang Q., Weber C., Schubert U.S., Hoogenboom R. (2017). Thermoresponsive Polymers with Lower Critical Solution Temperature: From Fundamental Aspects and Measuring Techniques to Recommended Turbidimetry Conditions. Mater. Horiz..

[4] Otake K., Inomata H., Konno M., Saito S. (1990). Thermal Analysis of the Volume Phase Transition with N-Isopropylacrylamide Gels. Macromolecules.

[5] Ksendzov E.A., Nikishau P.A., Zurina I.M., Presniakova V.S., Timashev P., Rochev Y.A., Kotova S., Kostjuk S.V. (2022). Graft Copolymers of N-Isopropylacrylamide with Poly(d,l-Lactide) or Poly(ε-Caprolactone) Macromonomers: A Promising Class of Thermoresponsive Polymers with a Tunable LCST. ACS Appl. Polym. Mater..

[6] Kurzbach D., Junk M.J.N., Hinderberger D. (2013). Nanoscale Inhomogeneities in Thermoresponsive Polymers. Macromol. Rapid Commun..

[7] Schmaljohann D. (2006). Thermo- and PH-Responsive Polymers in Drug Delivery. Adv. Drug Deliv. Rev..

[8] Cao M., Wang Y., Hu X., Gong H., Li R., Cox H., Zhang J., Waigh T.A., Xu H., Lu J.R. (2019). Reversible Thermoresponsive Peptide–PNIPAM Hydrogels for Controlled Drug Delivery. Biomacromolecules.

[9] Wu J.-Y., Liu S.-Q., Heng P.W.-S., Yang Y.-Y. (2005). Evaluating Proteins Release from, and Their Interactions with, Thermosensitive Poly (N-Isopropylacrylamide) Hydrogels. J. Control. Release.

[10] Agnihotri P., Sangeeta, Aery S., Dan A. (2021). Temperature- and PH-Responsive Poly(N-Isopropylacrylamide-Co-Methacrylic Acid) Microgels as a Carrier for Controlled Protein Adsorption and Release. Soft Matter.

[11] Kanazawa H., Okano T. (2011). Temperature-Responsive Chromatography for the Separation of Biomolecules. J. Chromatogr. A.

[12] Gao X., Cao Y., Song X., Zhang Z., Xiao C., He C., Chen X. (2013). PH- and Thermo-Responsive Poly(N-Isopropylacrylamide-Co-Acrylic Acid Derivative) Copolymers and Hydrogels with LCST Dependent on PH and Alkyl Side Groups. J. Mater. Chem. B.

[13] Efremov Y.M., Zurina I.M., Presniakova V.S., Kosheleva N.V., Butnaru D.V., Svistunov A.A., Rochev Y.A., Timashev P.S. (2021). Mechanical Properties of Cell Sheets and Spheroids: The Link between Single Cells and Complex Tissues. Biophys. Rev..

[14] Li M., Ma J., Gao Y., Yang L. (2019). Cell Sheet Technology: A Promising Strategy in Regenerative Medicine. Cytotherapy.

[15] Takezawa T., Mori Y., Yoshizato K. (1990). Cell Culture on a Thermo-Responsive Polymer Surface. Nat. Biotechnol..

[16] Nash M.E., Fan X., Carroll W.M., Gorelov A.V., Barry F.P., Shaw G., Rochev Y.A. (2013). Thermoresponsive Substrates Used for the Expansion of Human Mesenchymal Stem Cells and the Preservation of Immunophenotype. Stem Cell Rev. Rep..

[17] Heskins M., Guillet J.E. (1968). Solution Properties of Poly(N-Isopropylacrylamide). J. Macromol. Sci. Part A—Chem..

[18] Frolova A., Ksendzov E., Kostjuk S., Efremov Y., Solovieva A., Rochev Y., Timashev P., Kotova S. (2021). Thin Thermoresponsive Polymer Films for Cell Culture: Elucidating an Unexpected Thermal Phase Behavior by Atomic Force Microscopy. Langmuir.

[19] Ikada Y., Tsuji H. (2000). Biodegradable Polyesters for Medical and Ecological Applications. Macromol. Rapid Commun..

[20] Lee B.H., Vernon B. (2005). Copolymers OfN-Isopropylacrylamide, HEMA-Lactate and Acrylic Acid with Time-Dependent Lower Critical Solution Temperature as a Bioresorbable Carrier. Polym. Int..

[21] Tebong Mbah V., Pertici V., Lacroix C., Verrier B., Stipa P., Gigmes D., Trimaille T. (2020). A Sacrificial PLA Block Mediated Route to Injectable and Degradable PNIPAAm-Based Hydrogels. Polymers.

[22] Pertici V., Pin-Barre C., Rivera C., Pellegrino C., Laurin J., Gigmes D., Trimaille T. (2019). Degradable and Injectable Hydrogel for Drug Delivery in Soft Tissues. Biomacromolecules.

[23] Rana M.M., De la Hoz Siegler H. (2021). Tuning the Properties of PNIPAm-Based Hydrogel Scaffolds for Cartilage Tissue Engineering. Polymers.

[24] Mader K. (1996). Non-Invasive in Vivo Characterization of Release Processes in Biodegradable Polymers by Low-Frequency Electron Paramagnetic Resonance Spectroscopy. Biomaterials.

[25] Hinderberger D. (2011). EPR Spectroscopy in Polymer Science. EPR Spectrosc..

[26] Zubanova E.M., Kostjuk S.V., Timashev P.S., Rochev Y.A., Kokorin A.I., Melnikov M.Y., Golubeva E.N. (2021). Inhomogeneities in PNIPAM Aqueous Solutions: The Inside View by Spin Probe EPR Spectroscopy. Polymers.

[27] Kurzbach D., Schömer M., Wilms V.S., Frey H., Hinderberger D. (2012). How Structure-Related Collapse Mechanisms Determine Nanoscale Inhomogeneities in Thermoresponsive Polymers. Macromolecules.

[28] Caragheorgheopol A., Caldararu H., Dragutan I., Joela H., Brown W. (1997). Micellization and Micellar Structure of a Poly(Ethylene Oxide)/Poly(Propylene Oxide)/Poly(Ethylene Oxide) Triblock Copolymer in Water Solution, as Studied by the Spin Probe Technique. Langmuir.

[29] Caldararu H., Caragheorgheopol A., Dimonie M., Donescu D., Dragutan I., Marinescu N. (1992). Structure of Reversed Micelles In the Cyclohexane/Polyoxyethylene(4)Nonylphenol/Water System, As Studied by Spin Probe Technique. J. Phys. Chem..

[30] Soule B.P., Hyodo F., Matsumoto K., Simone N.L., Cook J.A., Krishna M.C., Mitchell J.B. (2007). The Chemistry and Biology of Nitroxide Compounds. Free Radic. Biol. Med..

[31] Kokorin A. (2012). Nitroxides—Theory, Experiment and Applications.

[32] Stoll S., Schweiger A. (2006). EasySpin, a Comprehensive Software Package for Spectral Simulation and Analysis in EPR. J. Magn. Reson..

[33] Schneider D.J., Freed J.H. (1989). Calculating Slow Motional Magnetic Resonance Spectra. Spin Labeling.

[34] Freed J.H., Fraenkel G.K. (1963). Theory of Linewidths in Electron Spin Resonance Spectra. J. Chem. Phys..

[35] Salikhov K.M. (2019). Current State of the Spin Exchange Theory in Dilute Solutions of Paramagnetic Particles. New Paradigm of Spin Exchange and Its Manifestations in EPR Spectroscopy. Phys.-Uspekhi.

[36] Ellison W.J. (2007). Permittivity of Pure Water, at Standard Atmospheric Pressure, over the Frequency Range 0–25 THz and the Temperature Range 0–100 °C. J. Phys. Chem. Ref. Data.

[37] Kecki Z., Łyczkowski B., Kołodziejski W. (1986). Critical Comparison of Empirical Systems Used to Describe Solvent Properties. J. Solution Chem..

[38] Dewez J.-L., Lhoest J.-B., Detrait E., Berger V., Dupont-Gillain C.C., Vincent L.-M., Schneider Y.-J., Bertrand P., Rouxhet P.G. (1998). Adhesion of Mammalian Cells to Polymer Surfaces: From Physical Chemistry of Surfaces to Selective Adhesion on Defined Patterns. Biomaterials.

[39] Dewez J.-L., Doren A., Schneider Y.-J., Rouxhet P.G. (1999). Competitive Adsorption of Proteins: Key of the Relationship between Substratum Surface Properties and Adhesion of Epithelial Cells. Biomaterials.

[40] García-Peñas A., Biswas C.S., Liang W., Wang Y., Yang P., Stadler F.J. (2019). Effect of Hydrophobic Interactions on Lower Critical Solution Temperature For. Polymers.

[41] Yang M., Liu C., Zhao K. (2017). Concentration Dependent Phase Behavior and Collapse Dynamics of PNIPAM Microgel by Dielectric Relaxation. Phys. Chem. Chem. Phys..

[42] Stoll S. (2014). Computational Modeling and Least-Squares Fittingof EPR Spectra. Handb. Multifrequency Electron. Paramagn. Reson. Data Tech..

[43] Lebedev Y.S., Grinberg O.Y., Dubinsky A.A., Poluektov O.G. (1992). Investigation of Spin Labels and Probes by Millimeter Band EPR. Bioactive Spin Labels.

[44] Bogdanov A.V., Vorobiev A.K. (2016). Orientation Order and Rotation Mobility of Nitroxide Biradicals Determined by Quantitative Simulation of EPR Spectra. Phys. Chem. Chem. Phys..

